# BMC Medical Imaging reviewer acknowledgement 2015

**DOI:** 10.1186/s12880-016-0119-y

**Published:** 2016-03-07

**Authors:** Clare Partridge

**Affiliations:** BioMed Central, Floor 6, 236 Gray’s Inn Road, London, WC1X 8HB UK

## Abstract

The editors of BMC Medical Imaging would like to thank all our reviewers who have contributed to the journal in Volume 15 (2015).

**Jawdat Abdulla**

Denmark

**Mariam Afshin**

Canada

**Walter Akers**

USA

**Toshihiro Akisue**

Japan

**Anthony Aletras**

Greece

**Dina Ibrahim Al-Sudani**

Saudi Arabia

**Jeff Anderson**

USA

**Neelapala Anil Kumar**

India

**Ulas Bagci**

USA

**Richard Barr**

USA

**Nicolau Beckmann**

Switzerland

**Ashwin Belle**

USA

**Simona Ben-Haim**

Israel

**Mikhail Berezin**

USA

**Tina Binderup**

Denmark

**Sotirios Bisdas**

Germany

**Piero Boraschi**

Italy

**Rasim Boyacıoğlu**

UK

**Steven Boyd**

Canada

**Giuseppe Brancatelli**

Italy

**Sarah Buehler**

Germany

**Marcus Carlsson**

Sweden

**Chandan Chakraborty**

India

**Claire Chalopin**

Germany

**Julia Chariker**

USA

**Jean Chen**

Canada

**Hairong Chen**

USA

**Yingyao Chen**

China

**Xiangyu Chen**

Singapore

**Woojin Cho**

USA

**James Cole**

UK

**Viviane Créteur**

Belgium

**Gillian Crofts**

UK

**Marco Daperno**

Italy

**Chandan J Das**

India

**Sudeb Das**

India

**Søren Daugaard**

Denmark

**Jie Deng**

USA

**Alen Docef**

USA

**Olov Duvernoy**

UK

**Lori Dwyer-Nield**

USA

**Armin Eilaghi**

Canada

**Ahmed El Kaffas**

USA

**Abdelaziz Elnekidy**

Egypt

**Kjersti Engan**

Norway

**Jan Engvall**

Sweden

**Kadir Erkmen**

USA

**Julio Urrutia Escobar**

Chile

**Hannu Eskola**

Finland

**Dimitrios Stergios Evangelopoulos**

Switzerland

**Zhaoyang Fan**

USA

**Troy Farncombe**

Canada

**Abiola Fasina**

UK

**Heather Faulkner**

USA

**Harvey Feigenbaum**

USA

**Martin Fiebich**

Germany

**Alex Förster**

Germany

**Dimitrios Fotiadis**

Greece

**Victor Frenkel**

USA

**Huazhu Fu**

Singapore

**Sebastiano Galantucci**

Italy

**Julio Gali**

Brazil

**Sshivanand Gamanagatti**

India

**Juan Pablo Gambini**

Uruguay

**Carlo Gandolfo**

Italy

**Yann Gavet**

France

**Pantelis Georgiadis**

Greece

**Oke Gerke**

Denmark

**Mahsa Ghaffari**

Iran

**Rossano Girometti**

Italy

**Gerhard Glatting**

Germany

**Ajit Goenka**

USA

**Ankur Goyal**

India

**Ersin Gunay**

Turkey

**Gang Guo**

China

**Maged Habib**

UK

**Mehdi Habibzadeh**

Canada

**Mark Haidekker**

USA

**Hirofumi Hanaoka**

Japan

**Samir Hanna**

Brazil

**Erik Hanson**

Norway

**Smriti Hari**

India

**Dennis Hedderich**

Germany

**Einar Heiberg**

Sweden

**Thomas Henzler**

Germany

**Soeren Hess**

Denmark

**Erlend Hodneland**

Norway

**Yue Huang**

China

**Martin Huellner**

Switzerland

**Toru Ishikawa**

Japan

**Robert Jablonowski**

Sweden

**Saurabh Jain**

Belgium

**Jeroen Jeneson**

Netherlands

**Soo Ji**

USA

**Lynne Jones**

USA

**Sven Jonuscheit**

UK

**Devasenathipathy Kandasamy**

India

**Eunji Kang**

USA

**Michael Kaplan**

USA

**Claude Kauffmann**

Canada

**Farzad Khalvati**

Canada

**Hyun Koo Kim**

South Korea

**Kyung Won Kim**

South Korea

**Despina Kontos**

USA

**Kamaleshwaran Koramadai**

India

**Atin Kumar**

India

**Yogesh Kumar**

USA

**Ngan Le**

USA

**Dong Ho Lee**

South Korea

**Yoon Jin Lee**

South Korea

**Young Han Lee**

South Korea

**Tim Leiner**

Netherlands

**Alexander Lembcke**

Germany

**Barrett Levesque**

USA

**Jianqiang Li**

China

**Huanguo Li**

China

**Ke Li**

USA

**Chunxia Li**

USA

**Kumble Madhusudhan**

India

**Smita Manchanda**

India

**Peter Mandl**

Austria

**Liu Manhua**

China

**Claudia Mani**

USA

**Fernando Marin**

Spain

**Joan Marti**

Spain

**Andrzej Materka**

Poland

**Kyung-San Min**

South Korea

**Nazanin Mirshahi**

UK

**Laurens Mitchell-Heggs**

France

**Hideki Mizu-Uchi**

Japan

**Raja Muthupillai**

USA

**Yoshinari Nagakane**

Australia

**Madhu Nair**

USA

**Takahito Nakajima**

Japan

**J Gail Neely**

USA

**Valentine Nfonsam**

USA

**Lori Nield**

USA

**Sarah Nordmeyer**

Germany

**Ken Okazaki**

Japan

**Catherine Oppenheim**

France

**Declan O'Regan**

UK

**Dustin Osborne**

USA

**Ellen Ostenfeld**

Sweden

**Yelda Ozsunar**

Turkey

**Markus Pääkkönen**

Finland

**John Pani**

USA

**Jijo Paul**

USA

**Xi Peng**

China

**Alessia Pepe**

Italy

**Ronald Peshock**

USA

**Antonius Pizanis**

Germany

**Prateek Prasanna**

USA

**Peter Proff**

Germany

**Xulei Qin**

USA

**Thomas Randau**

Germany

**Fabien Ricard**

France

**Matthias Rief**

Germany

**James Ritchie**

UK

**Patrik Rogalla**

Canada

**Peter Rogelj**

Slovenia

**Olivier Rouvière**

France

**Moussa Sacko**

Mali

**Umar Sadat**

UK

**Maythem Saeed**

USA

**M K Salih**

Saudi Arabia

**Shadrokh Samavi**

Iran

**Francesca Sanguedolce**

Italy

**Giulioa Santoro**

United Arab Emirates

**Mahesh Saqcena**

USA

**Pinaki Sarder**

USA

**Stephen Scharf**

USA

**Sabine Schmidt**

Switzerland

**Hugo Schnack**

Netherlands

**Ferdinand Schweser**

USA

**Anna Sedda**

Italy

**Parish Sedghizadeh**

USA

**Jing Shan**

USA

**Aparna Sharma**

India

**Sandra Shefelbine**

USA

**Clive Shiff**

USA

**Chris Sistrom**

USA

**Maxim Solovchuk**

Taiwan

**Francesco Somma**

Italy

**Geoffrey Sonn**

USA

**S M Reza Soroushmehr**

USA

**Steven Sourbron**

UK

**Rachel Sparks**

UK

**Shubhika Srivastava**

USA

**Sobczak Stéphane**

Belgium

**Yogesh Thakur**

Canada

**Marc Thiriet**

France

**Greg Thurber**

USA

**Johannes Töger**

Sweden

**Hiroyuki Tokue**

Japan

**Hana Dobsicek Trefna**

Sweden

**Hervé Trillaud**

France

**Du-Yih Tsai**

Japan

**Martin Ugander**

Sweden

**Matthew Urban**

USA

**Osslan Osiris Vergara Villegas**

Mexico

**Esther Verstraete**

Netherlands

**Anushya Vijayananthan**

Malaysia

**Parnpen Viriyavejakul**

Thailand

**Rozemarijn Vliegenthart**

Netherlands

**Surabhi Vyas**

India

**Justin Wan**

Canada

**Marc-André Weber**

Germany

**Friedrich Wetterling**

Ireland

**Susan Wiegers**

USA

**Timothy Wilson**

Canada

**Alexander Wong**

Canada

**Jie Wu**

USA

**Mengdi Xu**

Singapore

**Yanwu Xu**

Singapore

**Meng Yin**

UK

**Jeong Hee Yoon**

South Korea

**Zhicong Yu**

USA

**Xuyi Yue**

USA

**Xiaoyong Zhang**

USA

**Yufeng Zhou**

Singapore

**Alex Zijdenbos**

Canada

**Frank Zöllner**

Germany

